# Secreted Proteases Control Autolysin-mediated Biofilm Growth of Staphylococcus aureus*

**DOI:** 10.1074/jbc.M113.502039

**Published:** 2013-08-22

**Authors:** Chen Chen, Vengadesan Krishnan, Kevin Macon, Kartik Manne, Sthanam V. L. Narayana, Olaf Schneewind

**Affiliations:** From the ‡ Department of Microbiology, University of Chicago, Chicago, Illinois 60637,; the § Regional Centre for Biotechnology, Biotech Science Cluster, Gurgaon-122016, Haryana, India, and; the ¶ Center for Biophysical Sciences and Engineering, School of Optometry, University of Alabama at Birmingham, Birmingham, Alabama 35294

**Keywords:** Bacterial Pathogenesis, Biofilm, Extracellular Matrix, Serine Protease, Staphylococcus aureus

## Abstract

*Staphylococcus epidermidis*, a commensal of humans, secretes Esp protease to prevent *Staphylococcus aureus* biofilm formation and colonization. Blocking *S. aureus* colonization may reduce the incidence of invasive infectious diseases; however, the mechanism whereby Esp disrupts biofilms is unknown. We show here that Esp cleaves autolysin (Atl)-derived murein hydrolases and prevents staphylococcal release of DNA, which serves as extracellular matrix in biofilms. The three-dimensional structure of Esp was revealed by x-ray crystallography and shown to be highly similar to that of *S. aureus* V8 (SspA). Both *atl* and *sspA* are necessary for biofilm formation, and purified SspA cleaves Atl-derived murein hydrolases. Thus, *S. aureus* biofilms are formed via the controlled secretion and proteolysis of autolysin, and this developmental program appears to be perturbed by the Esp protease of *S. epidermidis*.

## Teaser

**Background:** Esp, a secreted protease of *Staphylococcus epidermidis*, blocks biofilm formation of *Staphylococcus aureus* and its ability to colonize human nares.

**Results:** Esp cleaves autolysin, thereby preventing the release of staphylococcal DNA as biofilm matrix.

**Conclusion:** Secreted proteases control *S. aureus* biofilm development and host colonization.

**Significance:** Methods that promote autolysin degradation may also prevent *S. aureus* colonization of humans.

## Introduction

*Staphylococcus aureus* is both a commensal and an invasive pathogen that causes skin and soft tissue infections, sepsis, and endocarditis (1). The primary niche for *S. aureus* colonization are the human nares (2). Approximately 20% of the human population is colonized persistently, whereas 30% represent intermittent carriers, and 50% are noncarriers (3). Nosocomial *S. aureus* bacteremia is three times more frequent in carriers than in noncarriers (3, 4). Colonization with highly virulent, multidrug-resistant strains (methicillin-resistant *S. aureus*, MRSA) is associated with invasive disease and treatment failure (5). *S. aureus* is currently the most frequent cause of infectious disease morbidity and mortality in the United States (6). Thus, strategies are needed to prevent *S. aureus* nasal colonization without selecting for antibiotic resistance and with the ultimate goal of reducing the incidence of staphylococcal infections.

Colonization of human nares is thought to involve the establishment of *S. aureus* biofilms (7). Work from many laboratories suggests that *S. aureus* biofilm growth occurs as a developmental program whereby bacteria initially adhere to host epithelial surfaces and subsequently release some of their DNA as extracellular matrix to replicate as biofilm communities (8, 9). Biofilm growth is also associated with the shedding of staphylococci, where released bacteria promote invasive disease or disseminate within host tissues (9). Several secreted products have been reported to function as adhesins for *S. aureus* biofilm formation, including fibronectin binding proteins (FnbA and FnbB) (10, 11), the extracellular adhesion protein (Eap) (12, 13), and the extracellular matrix protein (Emp) (13). *S. aureus* biofilms use bacterial DNA as an extracellular matrix (14, 15), which is released via Atl,^2^ a multifunctional murein hydrolase (16, 17). In addition to *atl*, the release of DNA by *S. aureus* grown in biofilms is also dependent on the *cidABC* and *lrgAB* operons, which appear to function as holins/antiholins by either initiating or preventing staphylococcal entry into a programmed cell death pathway (17). The expression of the *cid* and *lrg* operons is controlled in response to environmental signals via the LysR type regulator CidR and the two-component regulator LytRS, respectively (8, 18).

The 1256-residue autolysin precursor is secreted via its N-terminal signal peptide. Following signal peptide removal, pro-Atl is cleaved at two sites, residues 302 and 874, thereby generating the mature amidase (*N*-acetylmuramoyl-l-alanine amidase (AM, residues 303–874) and *N*-acetylglucosaminidase domains (GL, residues 875–1276)) (19). Each of the two enzymes is endowed with repeat domains (R1-R2-R3) that are tethered either to the C-terminal end of AM (R1-R2, residues 534–874) or the N-terminal end of GL (R3, residues 875–1016) (20). Each repeat domain folds into two half-open barrel subunits that bind polyglycerol-phosphate lipoteichoic acid at accessible sites in the bacterial envelope (21). Surface access is limited to peptidoglycan in the vicinity of the cell division septum (22, 23), because these sites are not occluded by polyribitol-phosphate wall teichoic acids (24). Deletion mutations in the *atl* gene abolish *S. aureus* biofilm formation, and *atl* mutants form large clusters of cells with incompletely separated cell wall envelopes (25).

Studying nasal colonization in human volunteers, Iwase *et al.* (7) observed a negative correlation between the colonization of *Staphylococcus epidermidis* strains expressing *esp* and *S. aureus*. Co-culturing of *S. epidermidis* strains expressing *esp* inhibited *S. aureus* biofilm formation (7). Although Esp does not affect the viability of *S. aureus*, the purified protease prevents biofilm formation and promotes disassembly of pre-established biofilms (7). Esp was found to degrade 75 different proteins in *S. aureus* biofilms (26). Nevertheless, previous work left unresolved by what mechanism Esp may interfere with *S. aureus* biofilms (26).

## EXPERIMENTAL PROCEDURES

### 

#### 

##### Bacterial Strains and Reagents

*S. aureus* Newman (27) and its variant with a *bursa aurealis* insertion in *atl* (28) have been previously described. The *atl* mutational lesion was transduced with bacteriophage ϕ85 into wild-type *S. aureus* Newman. Staphylococci were grown in tryptic soy broth (TSB) or on tryptic soy agar plates. Erythromycin (10 μg/ml) was used to select for the *bursa aurealis* insertional variant. *Escherichia coli* strains were grown in Luria broth or on Luria agar supplemented with ampicillin (100 μg/ml). Chemicals were purchased from Sigma unless indicated otherwise.

##### Esp Expression and Purification

Pro-Esp (Met^1^–Gln^282^) with an N-terminal His tag was cloned into pET28b, expressed in *E. coli* BL21 (DE3) cells and purified using nickel affinity chromatography (nickel-nitrilotriacetic acid Superflow agarose resin; Qiagen) (29). Mature Esp was purified by cleaving pro-Esp with thermolysin followed by gel filtration chromatography (Superdex 75 10/30 column; GE Healthcare) with 20 mm Tris-HCl (pH 7.2), 150 mm NaCl. Briefly, purified pro-Esp was incubated with thermolysin at 37 °C for 4 h, and cleavage was quenched by the addition of 5 mm EDTA. The purity and proteolytic activity of Esp were confirmed by SDS-PAGE and azocasein assay, respectively (29). Esp was concentrated to 22 mg/ml using an Amicon ultrafiltration system.

##### Esp Crystallization and Structure Determination

Concentrated, mature Esp was crystallized using the hanging drop vapor diffusion method (29). A droplet consisting of 1 μl of protein (22 mg ml^−1^ in 20 mm Tris-HCl, pH 7.2, 150 mm NaCl) and 1 μl reservoir solution (0.25 m potassium acetate, 22% PEG 3350) was equilibrated against 1 ml of reservoir solution at 22 °C. Native diffraction data were collected to 1.8 Å resolution on a R-AXIS IV imaging plate detector mounted on an in-house RIGAKU® rotating anode x-ray generator operating at 100 mA and 50 kV and using 20% (v/v) ethylene glycol as a cryoprotectant. Diffraction data were processed with D*TREK (30). The native Esp crystals belonged to the monoclinic space group P21 with one molecule in the asymmetric unit. Data collection and processing statistics are reported in Table 1.

**TABLE 1:** **Data collection, processing, and refinement statistics** Numbers in parentheses correspond to the values in the highest resolution shell.

	Esp
**Data collection**	
Resolution range (Å)	41.9–1.8 (1.86–1.8)
Space group	*P*2_1_

**Data processing**	
Unit cell parameters (*a*, *b*, *c* in Å; β in °)	39.4, 60.4, 42.3; 98.6
Unique reflections	17,810
Multiplicity	5.2 (5.1)
Mean *I*/σ*I* (*I*)	20.2 (5.9)
Completeness (%)	97.4 (95.1)
*R*_merge_ (%)*^a^*	3.9 (20.1)
Overall B factor from Wilson (Å^2^)	23.8

**Refinement**	
*R*_work_/*R*_free_ (%)	17.3/19.9
Average *B* value (Å^2^)	22.4
Root mean square deviation in bond lengths (Å)	0.02
Root mean square deviation in bond angles (°)	2.05
No. of protein/solvent atoms	1667/185
Residues in favored/allowed/disallowed regions in the Ramachandran plot (%)	83.1/16.9/0.0
Protein Data Bank code	4JCN

*^a^ R*_merge_ = Σ_hkl_Σ_j_ |*I*(*hkl*)_i_ − [*I*(*hkl*)]|/Σ_hkl_Σ_i_ *I*(*hkl*), where *I*(*hkl*) is the intensity of symmetry-related reflections, and [*I*(*hkl*)] is the average intensity over all observations.

The crystal structure of Esp was solved by molecular replacement, with the help of PHASER (31), implemented in the CCP4 suite (32), using the crystal structure of V8 protease (Protein Data Bank (PDB) entry 1QY6) (33) as a search model. Model building and refinement were completed with the help of COOT (34) and REFMAC (35). The final model consisted of 216 residues (Val^67^–Gln^282^), and the refinement converged with *R*_work_/*R*_free_ values 17.3/19.9% (Table 1). The final model is of good quality, reflected by an excellent Ramachandran plot, with all residues displaying backbone angles in the allowed regions. The quality of the final model was examined using COOT and PROCHECK (36) and deposited into the Protein Data Bank (code 4JCN).

##### Cleavage of GST-Atl Hybrids by Esp and V8

GST-AM_ΔR1R2_, GST-GL_ΔR3_, and GST-GL were purified as described previously (20). GST-AM was purified via a modified protocol (37). *E. coli* BL21 (DE3) harboring pGST-AM was grown in 2 liters of Luria broth at 37 °C to *A*_600_ 0.5, expression was induced with 1 mm of isopropyl β-d-thiogalactopyranoside, and culture was incubated for an additional 3 h at 30 °C. The cells were harvested by centrifugation, suspended in STE lysis buffer (10 mm Tris-HCl, pH 8.0, 150 mm NaCl, 1 mm EDTA, 100 μg/ml lysozyme), and incubated on ice for 15 min followed by the addition of 5 mm DTT and 1.5 mm PMSF. Sarkosyl was added to a final concentration of 2% (w/v). Bacteria were disrupted in a French pressure cell at 6,000 p.s.i. followed by centrifugation for 10 min at 13,000 × *g*. The supernatant was transferred to a new tube, Triton X-100 was added to a final concentration of 2%, and samples were incubated at room temperature for 30 min and loaded onto 1 ml of glutathione-Sepharose 4B column (GE Healthcare) pre-equilibrated with STE. The column was washed with 100 ml of STE buffer. GST-AM was eluted with 10 ml of 20 mm glutathione, 10% glycerol, 10 mm Tris-HCl (pH 8.0), 120 mm NaCl. Purified GST-Atl hybrids (5 μg of GST-AM, GST-AM_ΔR1R2_, GST-GL, or GST-GL_ΔR3_) were incubated with 400 nm Esp or V8 for 20 min at 37 °C. Samples were subjected to SDS-PAGE, and proteins were stained with Coomassie Brilliant Blue.

##### Biofilm Assays

Static biofilm assays were performed using a previously described protocol (38). Single *S. aureus* colonies were used to inoculate 2 ml of TSB with 0.2% glucose and incubated overnight at 37 °C with shaking (250 rpm). Cultures were diluted to *A*_600_ 0.05, and 200-μl aliquots were added to Costar 3596 96-well polystyrene plates (Corning, Lowell, MA) pretreated with 100 μl of 1 μg/ml human fibronectin (BD, Franklin Lakes, NJ) in PBS overnight at 4 °C. Plates were incubated at 37 °C with 5% CO_2_ for 24 h and washed with 200 μl of PBS twice. Washed samples were treated with 100 μl of ethanol for 2 min and then stained with 100 μl of 0.41% crystal violet in 12% ethanol for 2 min. Excess stain was removed by three washes with 200 μl PBS. The remaining crystal violet staining was solubilized with 100 μl of 95% ethanol for 10 min, and absorbance of 595-nm light was measured. The average absorbance values of media-only wells were subtracted from wells that had been inoculated with *S. aureus*.

For biofilm restoration experiments, 2.5 μg of purified GST-Atl hybrids were incubated with *S. aureus* Newman on fibronectin-coated microtiter plates at 37 °C with 5% CO_2_ for 24 h. For biofilm disassembly experiments, *S. aureus* and *S. epidermidis* biofilms were formed for 24 h. Purified Esp or V8 (2.5 μg) were added, and samples were incubated for 24 h at 37 °C. Biofilms were quantified as described above and analyzed with the Student's *t* test using GraphPad Prism version 5.0 for Windows (GraphPad Software, La Jolla, CA.)

##### Biofilm Substrates of Esp

*S. aureus* biofilms were formed during growth in iron-depleted CRPMI (RPMI 1640) medium. Culture medium was depleted of iron by batch incubation with 6% (w/v) Chelex 100 and then supplemented with 10% RPMI 1640 to provide trace amounts of divalent cations for growth (13). *S. aureus* overnight cultures in TSB were diluted to *A*_600_ 0.05, and 50 μl were added to FALCON 150-mm culture dishes coated with 1 μg/ml human fibronectin. Plates were incubated at 37 °C with 5% CO_2_ for 24 h and washed three times with 35 ml of PBS each. Biofilm was removed with a cell scraper, suspended in 1 ml of PBS, and incubated with 400 nm Esp or left untreated for 16 h at 37 °C with rotation. Biofilm samples were subsequently boiled in sample buffer, and proteins separated by 10–20% gradient SDS-PAGE and visualized with Coomassie Blue staining. Protein bands were excised and identified with liquid chromatography tandem mass spectrometry at the Taplin Biological Mass Spectrometry Facility (Harvard Medical School).

##### Extracellular DNA in Staphylococcal Biofilms

*S. aureus* biofilms were formed in Costar 12-well polystyrene plates with 12-mm glass coverslips that had been pretreated with 1 μg/ml human fibronectin. The wells were washed three times with PBS and stained with 1 μm SYTO 9/propidium iodide (PI) at room temperature for 20 min. The wells were washed with PBS, and the samples were fixed with 4% paraformaldehdye. Coverslips were mounted on glass slides and viewed via light microscopy. Microscopy and image acquisition were performed with the Olympus “live cell” DSU spinning disk inverted confocal microscopy (Integrated Microscopy Core Facility, The University of Chicago). Images were obtained using a 40× objective. Fluorescence intensities from 15 random fields were quantified using ImageJ software.

##### Peptidoglycan Cleavage Assay

*S. aureus* peptidoglycan was purified as described previously (39). Briefly, staphylococci were grown in 2 liters of TSB to *A*_600_ 0.6 and centrifuged, and bacteria were washed in water, suspended in 4% SDS, and boiled for 30 min. Detergent was removed by washing staphylococci extensively in water. Staphylococci were subjected to bead beating, glass beads were removed, and cell debris was sedimented by centrifugation. The extract was incubated with 100 μg/ml amylase for 2 h, followed first by the addition of 10 μg/ml DNase and 50 μg/ml RNase for 2 h and then by incubation with 100 μg/ml trypsin for 16 h at 37 °C. Peptidoglycan extracts were centrifuged, washed with water, suspended in 1% SDS, and boiled for 15 min to heat-inactivate all enzymes. Peptidoglycan was extensively washed with water to remove all traces of SDS, followed by washing with 8 m LiCl, 100 mm EDTA, and acetone. The cell walls were then washed with water and lyophilized. Hydrofluoric acid was added and incubated for 48 h at 4 °C to remove teichoic acid. Peptidoglycan was neutralized, and sedimented murein sacculi were treated with alkaline phosphatase for 16 h at 37 °C. Purified peptidoglycan was boiled for 5 min, washed with water, and stored at 4 °C. Peptidoglycan was incubated with 5 μg of purified GST-AM, GST-AM_ΔR1R2_, GST-GL, or GST-GL_ΔR3_ in 0.1 m phosphate buffer (pH 7.0) for 16 h at 37 °C. Peptidoglycan cleavage was determined by measuring the *A*_600_ before and after incubation.

##### Staphylococcal Cell Cluster Analysis

Overnight cultures of *S. aureus* were diluted 1:100 in 100 μl of TSB and added to 96-well plates at 37 °C with shaking. Culture growth was monitored by reading *A*_600_ at 30-min intervals. Overnight cultures were diluted 1:100 in 1 ml of TSB and incubated at 37 °C for 2 h with shaking with or without 25 μg of GST-Atl hybrids. Cultures were subsequently centrifuged at 7,000 × *g* for 1 min and fixed with 4% paraformaldehyde prior to washing and suspension in 1 ml of PBS. Staphylococci were then analyzed at the University of Chicago flow cytometry facility using BD LSRII-Blue flow cytometer to measure the cluster size of *S. aureus* cells.

##### Activity Measurements of Staphylococcal Proteases

Conditioned extracellular media, obtained as the supernatant following centrifugation of overnight cultures of *S. aureus,* were concentrated 15-fold using the Amicon ultrafiltration system. Concentrated culture media in 20-μl aliquots were incubated with 480 μl of reaction mixture containing 1% azocasein, 100 mm Tris-HCl (pH 8.0) at 37 °C overnight. Following incubation, 25 μl of 100% TCA was added to quench each reaction; following centrifugation at 15,000 × *g* for 10 min, soluble material was recovered with the supernatant, and absorbance at 440 nm was measured to determine protease activity.

## RESULTS

### 

#### 

##### Esp Cleaves Atl in Staphylococcal Biofilms

**FIGURE 1.**
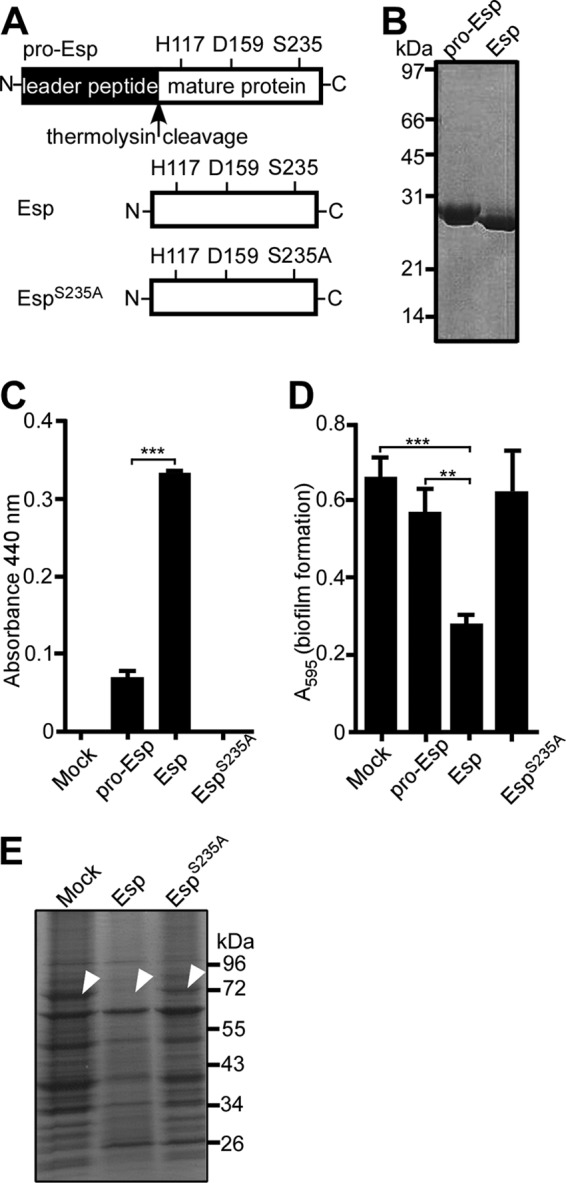
**Purified, recombinant Esp displays protease activity, inhibits *S. aureus* biofilm formation and cleaves Atl.** *A*, diagram illustrating the primary structure of pro-Esp, Esp, and the variant Esp^S235A^ that were purified from *E. coli*. The *arrow* indicates the thermolysin cleavage site. *B*, purified pro-Esp and Esp were separated by SDS-PAGE and stained with Coomassie Blue. *C*, Esp activity assay using azocasein substrate and measuring product absorbance at 440 nm. Enzyme activity measurements were averaged from three independent determinations, and the standard error of the means was determined (*brackets*). Statistical significance was determined with the two-tailed Student's *t* test. ***, *p* < 0.0001. *D*, purified pro-Esp, Esp, or Esp^S235A^ was incubated with *S. aureus* Newman during assembly of biofilms on fibronectin-coated microtiter plates at 37 °C with 5% CO_2_ over 24 h. Following incubation, the plates were washed and stained with crystal violet to measure biofilm formation as absorbance at 595 nm. Biofilm data were averaged from three independent determinations, and the standard error of the means was calculated (*brackets*). Statistical significance was assessed with the two-tailed Student's *t* test in pairwise comparison with mock treated samples. ***, *p* < 0.0001; **, *p* < 0.001. *E*, mock, Esp, or Esp^S235A^ treated *S. aureus* Newman biofilms were dispersed, and proteins were analyzed by Coomassie-stained SDS-PAGE. Protein species that were absent in Esp treated samples were identified by LC-MS/MS. *Arrows* identify the migratory position of Atl (AM).

Following signal peptide cleavage, the pro-form of Esp (pro-Esp) is cleaved in the extracellular medium of *S. epidermidis* cultures to generate mature Esp protease, which mediates the disassembly of *S. aureus* biofilms (7). We expressed six-histidyl-tagged pro-Esp in *E. coli* and purified recombinant protein by affinity chromatography (Fig. 1*A*). Thermolysin cleavage and gel filtration chromatography were used to obtain purified Esp (Fig. 1*B*). The variant Esp^S235A^ harbors an alanyl substitution at the active site serine residue of Esp (Fig. 1*A*). When examined for protease activity with azocasein substrate (40), Esp cleaved significantly more substrate than pro-Esp, whereas Esp^S235A^ did not display protease activity (Fig. 1*C*). Wild-type *S. aureus* strain Newman was used to form staphylococcal biofilms using human fibronectin as a matrix, which were quantified by crystal violet staining (41). Treatment with Esp, but not pro-Esp or Esp^S235A^, triggered disassembly of staphylococcal biofilms (Fig. 1*D*). Proteins in biofilms with or without Esp treatment were separated by 10–20% gradient SDS-PAGE, stained with Coomassie Brilliant Blue, and identified via LC-MS/MS (Fig. 1*E*). Treatment with Esp, but not Esp^S235A^, caused Atl degradation (Fig. 1*E*). Our experiments revealed that Esp cleaved 18 additional polypeptides, including FnbA, FnbB, Eap, and SpA, that had previously been identified as Esp substrates (26) (Table 2). Although the genes for some of these secreted proteins contribute to *S. aureus* biofilm formation, they are not essential for this developmental process. Of note, in *S. aureus* Newman biofilms, Atl is a highly abundant component and effectively degraded during Esp treatment (Fig. 1*E*). Considering the importance of Atl in biofilm development, we focused our experimental approach on the interactions between Esp and Atl.

**TABLE 2:** **Esp substrates in the S. aureus Newman biofilm**

Protein	Unique peptides/total	Description
Atl	56/116	Autolysin
Coa	51/209	Coagulase
Geh	46/104	Lipase
SpA	40/104	Protein A
Eap	38/138	Extracellular adherence protein
AM	35/87	Amidase
FnbA	34/94	Fibronectin-binding protein A
LukS	25/36	Leukocidin S
SdrE	24/31	Ser-Asp repeat protein E
ClpC	22/27	Clp protease subunit C
HlgA	21/46	γ-Hemolysin subunit A
Sbi	19/29	Staphylococcal binder of IgG
LukF	17/34	Leukocidin F
HlgB	16/35	γ-Hemolysin subunit B
FnbB	13/17	Fibronectin-binding protein B
Hla	13/16	α-Hemolysin
ClfB	12/14	Clumping factor B
HlgC	11/15	γ-Hemolysin subunit C
ClfA	9/15	Clumping factor A

##### Esp Treatment of Biofilms Formed from atl Staphylococci

**FIGURE 2.**
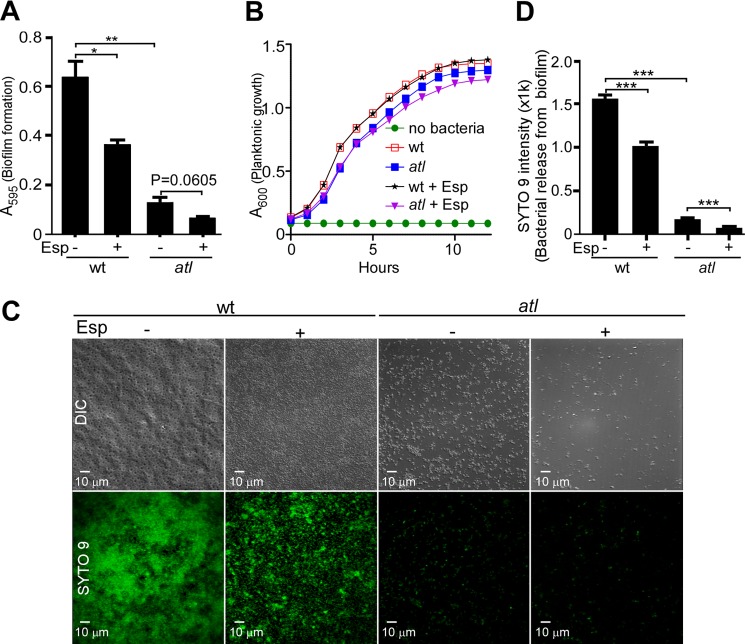
**Esp treatment of *S. aureus* Newman wild-type and *atl* mutant biofilms.** *A*, purified Esp or mock treatment were added during *S. aureus* wild-type (*wt*) or *atl* mutant biofilm assembly on fibronectin-coated microtiter plates at 37 °C with 5% CO_2_ for 24 h. Following incubation, plates were washed and stained with crystal violet to measure biofilm formation as absorbance at 595 nm (*A*_595_). Biofilm data were averaged from three independent determinations. The standard error of the means is indicated as *brackets*. Statistical significance was assessed with the two-tailed Student's *t* test. **, *p* < 0.001; *, *p* < 0.05. *B*, mock or 25 μg/ml Esp were added to tryptic soy broth inoculated with *S. aureus* Newman wt or *atl* mutant strains, incubated with rotation at 37 °C and growth measured via absorbance at 600 nm (*A*_600_). *C*, purified Esp or mock treatment was added during *S. aureus* Newman wt or *atl* mutant biofilm assembly on fibronectin-coated microtiter plates at 37 °C with 5% CO_2_ for 24 h. Plates were washed, viable staphylococci were stained with SYTO 9, and fluorescence was captured via fluorescence microscopy. *D*, differential interference contrast (*DIC*) and fluorescence microscopy images acquired in *C* were quantified with ImageJ. The data were averaged from three independent determinations. The standard error of the means is indicated as *brackets*. Statistical significance was assessed with the two-tailed Student's *t* test. ***, *p* < 0.0001.

Mutations in the autolysin gene (*atlE*) of *S. epidermidis* cause a dramatic reduction in biofilm formation (42). AtlE was initially shown to function as a *S. epidermidis* adhesin (purified AtlE binds host vitronectin, fibronectin, and Hsc70 receptor (42)). More recent work highlighted the contribution of *atl* in *S. aureus* UAMS-1, BH1CC, and many other MSSA and MRSA isolates toward releasing DNA as an extracellular matrix for staphylococcal biofilm formation (16, 38). This discovery was accompanied by the insight that *S. aureus*, but presumably not *S. epidermidis* (43), forms biofilms *in vitro* and *in vivo* without the *icaABCD* locus (16), which provides for the synthesis of (β1–6)-poly-*N*-acetylglucosamine exo-polysaccharide (44). We wondered whether *atl* mutant *S. aureus* Newman can form biofilms and, if so, whether *atl* bacterial communities can be disassembled by treatment with Esp. Compared with wild-type staphylococci, the *atl* mutant formed only a rudimentary biofilm that, when subjected to treatment with Esp, did not show significant disassembly (Fig. 2*A*). As a control, Esp treatment caused disassembly of biofilms formed by *S. aureus* Newman. When subjected to growth assays with rotating cultures, the *atl* mutant replicated at a rate indistinguishable from that of wild-type staphylococci (Fig. 2*B*), although the *atl* mutants formed large clusters of incompletely separated staphylococci (23) (see below). The growth of wild-type and *atl* mutant staphylococci was not perturbed when cultures were treated with Esp, indicating that protease treatment kills neither wild-type nor mutant strains (Fig. 2*B*). As a measure for the direct dispersal of bacteria from biofilms, staphylococci were labeled with SYTO9 and then subjected to Esp treatment. Esp released approximately half of wild-type staphylococci from biofilms and almost all *atl* bacteria from their rudimentary biofilm (Fig. 2, *C* and *D*). These experiments identify Atl as a key target of Esp, whose degradation prevents biofilm formation and is associated with the disassembly of biofilms. Moreover, the contributions of other targets of Esp, with known auxiliary functions in biofilm formation or stability (Eap, Emp, SpA, FnbA, and FnbB), are revealed as protease treatment eliminates the rudimentary biofilm of *atl* mutants.

##### Esp Cleavage of Atl

**FIGURE 3.**
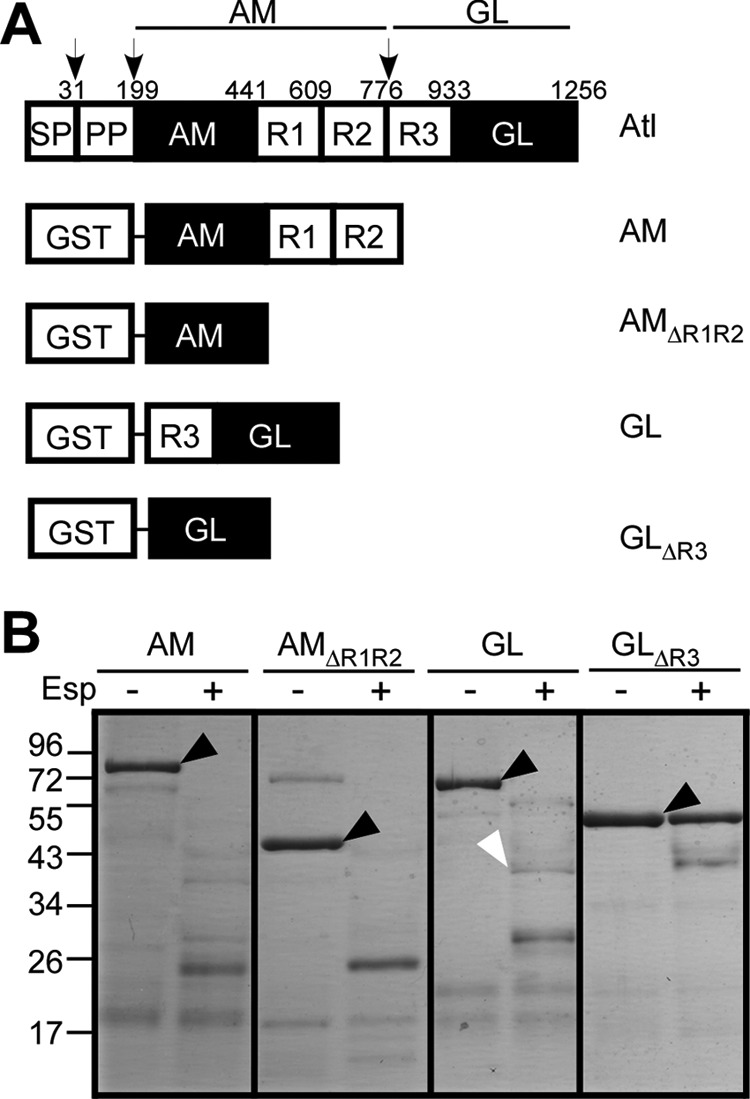
**Esp treatment of GST-Atl hybrids.** *A*, diagram illustrating the primary structure of GST hybrids with Atl domains including GST-AM, GST-AM_ΔR1R2_, GST-GL, and GST-GL_ΔR3_. *B*, purified GST hybrids (5 μg) were incubated with 400 nm Esp (+) or mock treated (−) for 20 min at 37 °C. Proteins were separated on SDS-PAGE followed by Coomassie Blue staining. *Black arrowheads* identify the migratory positions of GST-AM, GST-AM_ΔR1R2_, GST-GL, and GST-GL_ΔR3_. The *white arrowhead* identifies an Esp cleavage species of GST-GL, which had been cut after glutamyl 862 (E/VKTTQK), as identified by Edman degradation.

To determine which of the functional domains of Atl are cleaved by Esp, we purified AM (*N-*acetylmuramoyl-l-alanine amidase), AM_ΔR1R2_ (lacking the C-terminal repeat domains R1 and R2 of AM), GL (*N*-acetylglucosamine-*N*-acetylmuramic acid glucosaminidase), and GL_ΔR3_ (lacking the N-terminal R3 domain of GL) as hybrids fused to the C-terminal end of GST (Fig. 3. Esp treatment cut AM, AM_ΔR1R2_, and GL, but not GL_ΔR3_ (Fig. 3*B*). Esp treatment generated several cleavage fragments from AM, AM_ΔR1R2_, or GL, suggesting that the protease can cut at multiple sites within the amidase and the R1-R3 domains (Fig. 3*B*). Edman degradation of cleaved peptides identified glutamic acid residues (for example Glu^862^ in GL) as Esp cleavage sites (Fig. 3*B*).

##### Murein Hydrolase Activities of Esp-treated Atl

**FIGURE 4.**
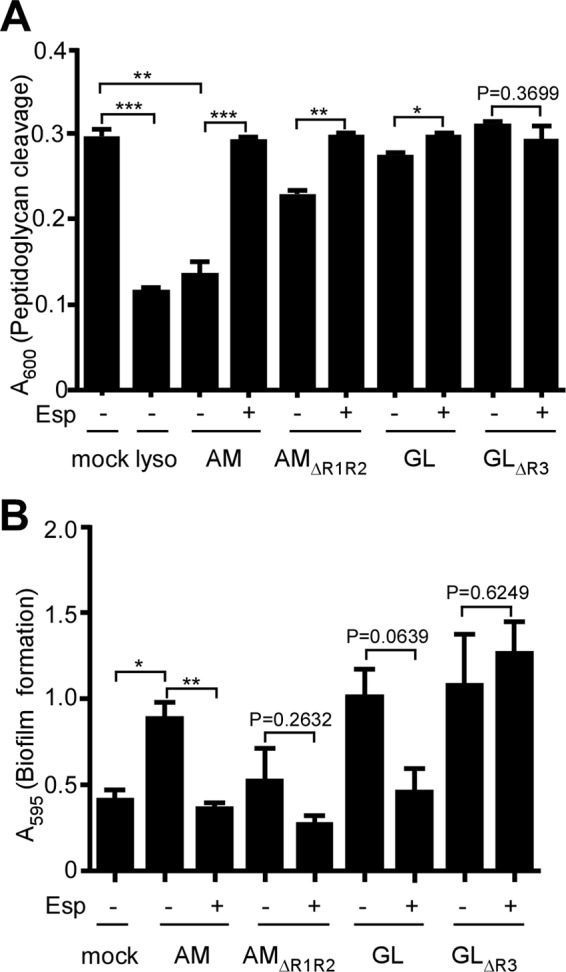
**Peptidoglycan hydrolase and biofilm promoting activity of GST-Atl hybrids in the presence or absence of Esp treatment.** *A*, *S. aureus* Newman murein sacculi were obtained with a bead beater instrument and extracted with detergent as well as hydrofluoric acid to remove membranes and wall teichoic acids, respectively. Cleavage of peptidoglycan by 5 μg of purified lysostaphin (*lyso*), GST-AM (*AM*), GST-AM_ΔR1R2_ (*AM_ΔR1R2_*), GST-GL (*GL*), or GST-GL_ΔR3_ (*GL_ΔR3_*) in the presence (+) or absence (−) of 400 nm Esp was monitored by measuring absorbance at 600 nm (*A*_600_). The data represent averages of three independent experimental determinations, and the standard error of the means is indicated by *brackets*. Statistical significance was assessed in pairwise comparison with the two-tailed Student's *t* test. ***, *p* < 0.0001; **, *p* < 0.001; *, *p* < 0.05). *B*, biofilm formation of the *atl* mutant on fibronectin-coated microtiter plates at 37 °C with 5% CO_2_ for 24 h was analyzed in the presence or absence (mock) of 5 μg of purified GST-AM (*AM*), GST-AM_ΔR1R2_ (*AM_ΔR1R2_*), GST-GL (*GL*), GST-GL_ΔR3_ (*GL_ΔR3_*), or 400 nm Esp (+ or −). Following incubation, the plates were washed and stained with crystal violet to measure biofilm formation as absorbance at 595 nm (*A*_595_). Biofilm data were averaged from three independent determinations. The standard error of the means is indicated as *brackets*. Statistical significance was assessed with the two-tailed Student's *t* test. **, *p* < 0.001; *, *p* < 0.05.

To explore the effects of Esp treatment on Atl murein hydrolase activities, we purified murein sacculi from wild-type *S. aureus* and extracted wall teichoic acids via hydrofluoric acid treatment (45). The murein hydrolase activities of AM and AM_ΔR1R2_ as well as GL and GL_ΔR3_ were determined by incubating GST hybrids with murein sacculi while monitoring absorbance at 600 nm. Similar to lysostaphin (46), a glycyl-glycine endopeptidase that cleaves staphylococcal cell wall cross-bridges (47), GST-AM treatment of peptidoglycan caused a large decrease in absorbance (Fig. 4*A*). Esp treatment abolished all peptidoglycan hydrolase activity of AM (Fig. 4*A*). Removal of the R1-R2 repeat domains of AM reduced the peptidoglycan hydrolase activity of AM_ΔR1R2_; however, this activity was also abolished by treatment with Esp (Fig. 4*A*). Finally, GL displayed very little activity in reducing the absorbance at 600 nm, which can be explained by the relatively short glycan chains and intricate cross-linking (>99%) of the staphylococcal cell wall (20). The murein hydrolase activity of GL was abolished by Esp treatment (Fig. 4*A*). GL_ΔR3_, which is a poor substrate for Esp, did not display murein hydrolase activity in this assay (Fig. 4*A*). Our results corroborate earlier observations on the genetic requirements of the AM and GL domains for *S. aureus* Atl function (38).

##### trans-Complementation of atl Mutant Biofilms

The addition of purified GST-AM or GST-GL restored (*trans*-complementation) the biofilm formation defect of *atl* mutant *S. aureus* Newman grown on fibronectin-coated microtiter plates (Fig. 4*B*). This activity was abolished following treatment of GST-AM with Esp (Fig. 4*B*). Esp treatment did not affect peptidoglycan hydrolase activity of GST-GL and did not affect GST-GL biofilm *trans*-complementation either. GST-AM_ΔR1R2_ also did not display biofilm *trans*-complementation for *atl* mutants, and Esp treatment did not affect this phenotype (Fig. 4*B*). GST-GL_ΔR3_ did not degrade murein sacculi and did not *trans*-complement the *atl* mutant biofilm defect (Fig. 4*B*). Esp treatment did not affect biofilm formation in the presence of GST-GL_ΔR3_ (Fig. 4*B*).

##### Esp Treatment and Staphylococcal Cell Clusters

**FIGURE 5.**
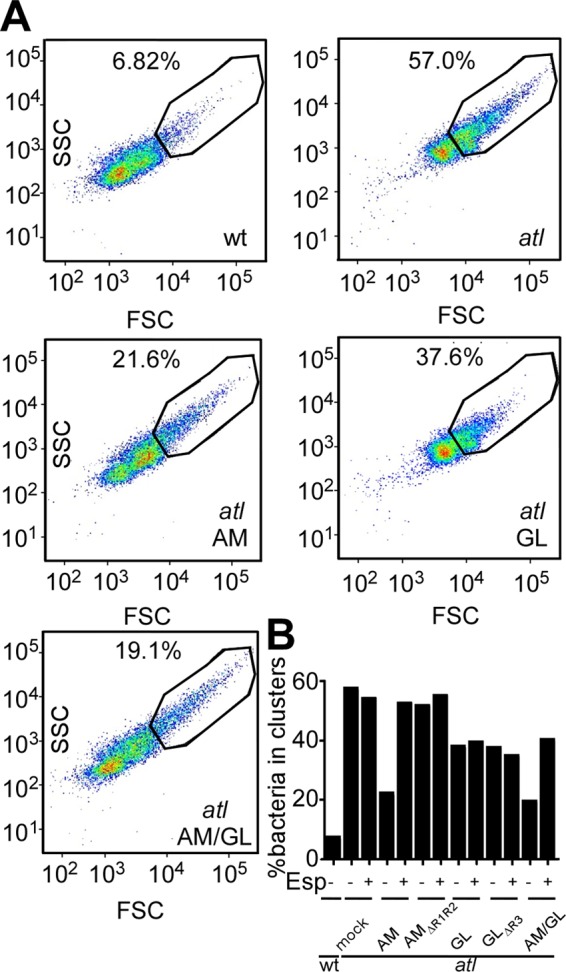
**Effect of Esp treatment on the staphylococcal cluster dispersing activity of GST-Atl hybrids.** *A*, overnight cultures of *S. aureus* Newman wild-type (*wt*) or *atl* variant were diluted to *A*_600_ 0.05 in 1 ml of TSB and incubated at 37 °C for 2 h in the presence or absence of 25 μg of purified GST-AM (*AM*), GST-AM_ΔR1R2_ (*AM_ΔR1R2_*), GST-GL (*GL*), GST-GL_ΔR3_ (*GL_ΔR3_*), or 400 nm Esp (+ or −). Staphylococci were fixed with 4% paraformaldehyde, washed, suspended with 1 ml of PBS, and analyzed by flow cytometry. *B*, percentage of bacteria in large cell clusters were quantified for wild-type and *atl* mutant staphylococci with or without Atl hybrids and Esp treatment.

The *atl* mutant staphylococci are defective in the separation of daughter cells following cell division, which is due to the incomplete separation of cross-wall peptidoglycan (23, 25). This phenotype can be quantified by flow cytometry, which revealed that 6.82% of wild-type but 57% of *atl* mutants exist as large cell clusters (Fig. 5*A*). Treatment with Esp did not affect the cell cluster size of *atl* mutants, whereas GST-AM, GST-GL, and GST-GL_ΔR3_, but not GST-AM_ΔR1R2_, reduced the cell cluster size (Fig. 5). The cluster reducing activity of GST-AM and GST-GL was abolished by treatment with Esp (Fig. 5).

##### Esp Treatment and Staphylococcal Release of Extracellular DNA

**FIGURE 6.**
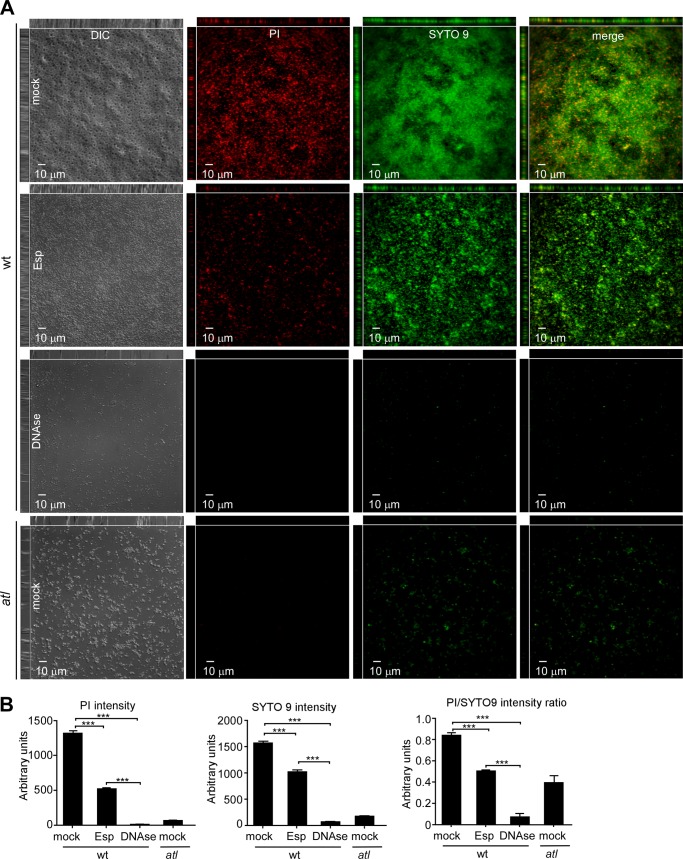
**Esp treatment and the release of extracellular DNA in *S. aureus* biofilms.** *A*, purified 400 nm Esp or DNase I were incubated with *S. aureus* Newman wild-type (*wt*) or its *atl* variant during biofilm assembly on fibronectin-coated microtiter plates at 37 °C with 5% CO_2_ for 24 h. Following incubation, plates were washed and stained with PI to reveal extracellular DNA or SYTO 9 to reveal viable staphylococci and analyzed via DIC and fluorescence microscopy. *DIC*, differential interference contrast. *B*, fluorescence intensity staining of PI, SYTO 9, or PI/SYTO 9 staining in samples from *A* was quantified with ImageJ. The data were averaged from three independent determinations, and the standard error of the means is indicated as *brackets*. Statistical significance was assessed in pairwise comparison using the two-tailed Student's *t* test. ***, *p* < 0.0001; **, *p* < 0.001; *, *p* < 0.05.

We sought to test the hypothesis that Esp blocks the release of extracellular DNA during biofilm formation and added the protease to either wild-type or *atl* mutant *S. aureus* Newman. Biofilms were stained with either PI as a measure for extracellular DNA or with SYTO9 to quantify viable staphylococci (Fig. 6). *S. aureus* biofilms harbored large amounts of extracellular DNA and bacterial cells, as quantified by PI and SYTO9 staining (Fig. 6). Treatment with Esp reduced the amount of extracellular DNA and viable staphylococci in wild-type biofilms, whereas treatment with DNase abolished both (Fig. 6). Rudimentary biofilms formed by *atl* mutant *S. aureus* harbored very little if any extracellular DNA and few staphylococci (Fig. 6).

##### SspA (V8 Protease) Is Required for S. aureus Biofilm Formation

**FIGURE 7.**
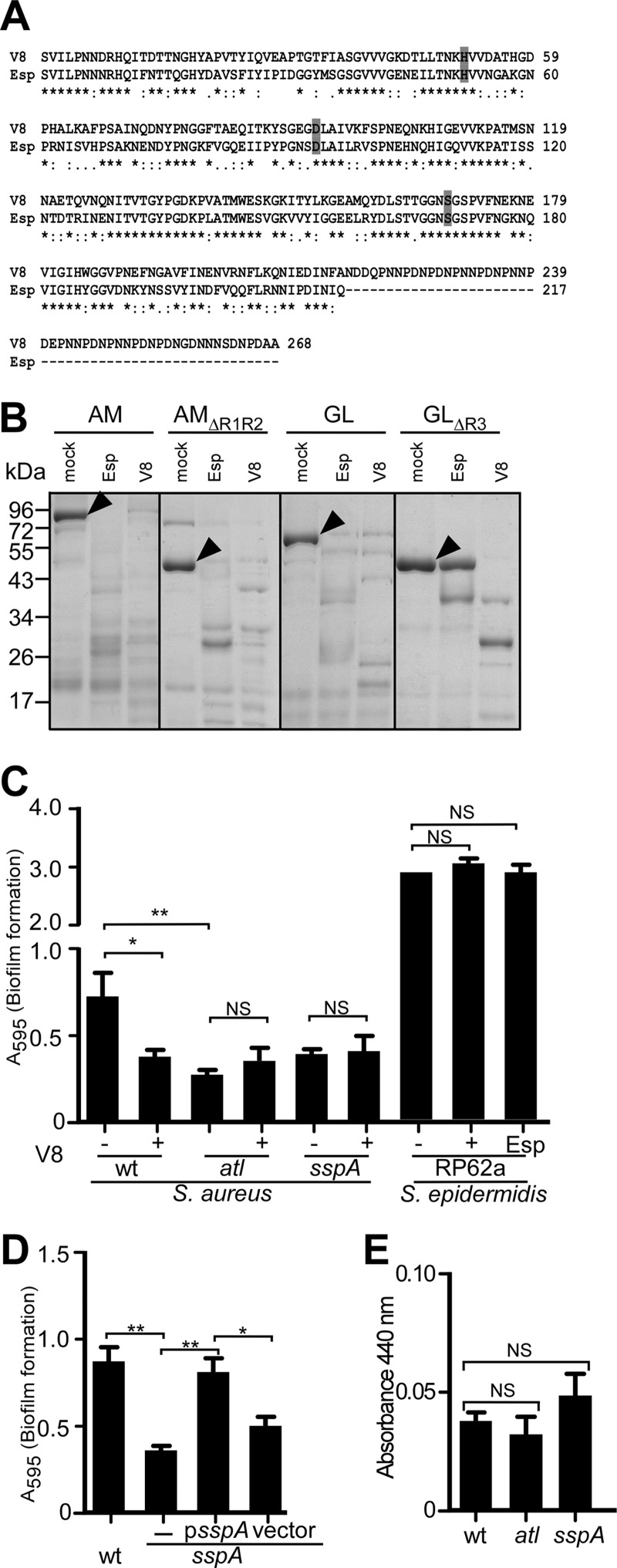
***S. aureus* V8 protease and biofilm formation.** *A*, protein sequence alignment of mature Esp and V8. *B*, GST-AM, GST-AM_ΔR1R2_, GST-GL, and GST-GL_ΔR3_ (5 μg) were incubated with 400 nm Esp (Esp), V8 protease (V8), or mock treatment (−) for 20 min at 37 °C. Proteins were separated on SDS-PAGE followed by Coomassie Blue staining. *Arrowheads* identify the migratory positions of GST-AM, GST-AM_ΔR1R2_, GST-GL, and GST-GL_ΔR3_. *C*, purified Esp, V8, or mock treatment were added during biofilm formation of *S. epidermidis* RP62a and *S. aureus* Newman wild-type and *atl* and *sspA* mutant strains on fibronectin-coated microtiter plates at 37 °C with 5% CO_2_ for 24 h. Following incubation, plates were washed and stained with crystal violet, and biofilm formation was measured as absorbance at 595 nm. *D*, *S. aureus* Newman wild-type (*wt*) or its *sspA* mutant without plasmid (−) or with *psspA* or vector control (pWW412) was incubated on fibronectin-coated microtiter plates at 37 °C with 5% CO_2_ for 24 h. Following incubation, the plates were washed and stained with crystal violet to measure biofilm formation as absorbance at 595 nm (*A*_595_). Biofilm data were averaged from three independent determinations. The standard error of the means is indicated as *brackets*. Statistical significance was assessed with the two-tailed Student's *t* test. **, *p* < 0.001; *, *p* < 0.05; *NS*, no significant difference. *E*, the activity of extracellular proteases secreted by *S. aureus* wild-type (*wt*), *atl* and *sspA* mutant cultures were quantified with the azocasein assay, and product cleavage was measured as absorbance at 440 nm. The protease activity data were averaged from three independent determinations. The standard error of the means is indicated as *brackets*. Statistical significance was assessed with the two-tailed Student's *t* test. *NS*, no significant difference.

V8 (GluC) enzyme is a serine protease that selectively cleaves peptides C-terminal of glutamyl or aspartyl residues (48, 49). *S. aureus* V8 is highly homologous to *S. epidermidis* Esp (59% identity and 78% similarity), although V8 harbors a C-terminal extension of 52 amino acids that is absent in Esp (Fig. 7*A*). Similar to Esp, purified V8 cleaved GST-AM, GST-AM_ΔR1R2_, and GST-GL (Fig. 7*B*). In contrast to Esp, the V8 enzyme effectively cleaved GST-GL_ΔR3_ (Fig. 7*B*). This can be explained by the protease activity of Esp, which, unlike V8, cuts only at the C-terminal of glutamyl but not of aspartyl (50, 51). To analyze the contribution of *sspA* toward *S. aureus* biofilm formation, we transduced a *bursa aurealis* insertional lesion in the *sspA* gene (28) into the wild-type strain Newman (27). When analyzed for biofilm formation on fibronectin matrix, the *sspA* mutant was significantly impaired, as compared with wild type and similar to the biofilm defect of the *atl* mutant (Fig. 7*C*). The culture supernatant of the *S. aureus* Newman *sspA* mutant did not display increased protease activity in the azocasein assay when compared with wild-type (Fig. 7*E*). Thus, the biofilm phenotype of the *sspA* mutant is not due to an increase in extracellular protease activity, as has been reported for the *sspA* deletion mutant of *S. aureus* SH1000 (52). Moreover, the biofilm phenotype of the *S. aureus* Newman *sspA* mutant was restored following transformation with a recombinant plasmid expressing wild-type *sspA*, but not with vector (pWW412) control (Fig. 7*D*). Incubation of *S. aureus* Newman with purified V8 protease reduced biofilm formation (Fig. 7*C*). V8 treatment did not improve biofilm formation of *S. aureus* Newman *atl* or *sspA* mutants (Fig. 7*C*). These results suggest that the expression and/or activity of secreted V8 protease must be carefully controlled during *S. aureus* biofilm formation, because treatment with exogenous, active V8 protease cannot complement the *sspA* mutant phenotype (Fig. 7*C*). Of note, neither V8 nor Esp protease treatment affected biofilm formation of *S. epidermidis* RP62a (Esp^+^), suggesting that the biofilm program of this microbe is not controlled by secreted serine proteases or their protease-sensitive substrates (Fig. 7*C*).

##### Crystallographic Structure of Esp

**FIGURE 8.**
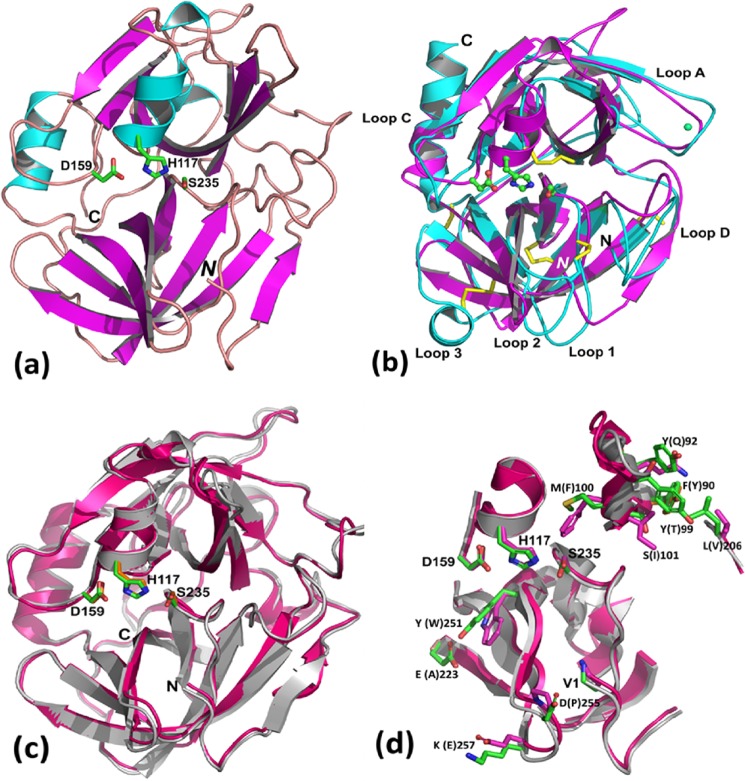
**Comparison between Esp and V8 crystal structures.** *a*, ribbon representation of the refined crystal structure of active Esp. The α-helices are represented in *cyan*, β-strands are in *magenta*, and loop regions are in *light brown*. The putative catalytic His^117^, Asp^159^, and Ser^235^ residue side chains are represented as *green sticks. b*, superposition of Esp (PDB code 4JCN, represented in *magenta*) and pancreatic trypsin (PDB code 1TRM, in *cyan*) crystal structures. Surface loops (A, C, D, 1, 2, and 3) that dictate substrate specificity for trypsin are labeled, and the disulfide bonds (in *yellow*) that hold its structure together are also shown. *c*, superposition of Esp (*magenta*) and V8 (*ivory*, PDB code 1QY6) crystal structures. *d*, significant residue differences observed between Esp (*magenta*) and V8 (*ivory*) crystal structures are shown in their respective positions. Side chains are shown as *sticks*: *green* for V8 and *magenta* for Esp residues.

Purified Esp was crystallized, and its three-dimensional structure was determined using x-ray crystallography. Esp displays a β-barrel fold assembled from two discrete domains and a C-terminal α-helix, similar to eukaryotic serine proteases of the chymotrypsin family (Fig. 8, *a* and *b*) (53–55). Even though Esp exhibits a highly conserved, compact β-barrel fold, the five or more intradomain disulfide bonds that are responsible for the structural rigidity of eukaryotic serine proteases are absent (54). Each of the two Esp domains is comprised of six antiparallel β-strands, and the solvent-accessible catalytic and substrate binding sites are situated at the interface of the two domains. The N-terminal domain (chymotrypsin nomenclature) is comprised primarily of residues Gln^77^–Ile^183^, whereas the C-terminal domain encompasses Ser^184^–Ile^264^. Although the position of the C-terminal α-helix (Asn^266^–Ile^276^) is conserved with that of other serine proteases, the N-terminal segment (Val^67^–Gln^76^) contains a short β-strand that is associated with the substrate-binding S1 pocket and distinct from eukaryotic serine proteases (Fig. 8*b*). In addition to the conserved position of putative catalytic triad residues (Ser^235^, Asp^159^, and His^117^), the substrate-binding region (S1 pocket) and the oxyanion hole, which together constitute the critical functional elements of activated serine proteases, are also conserved in Esp (Fig. 8*a*). A search for structural homologues of Esp identified *S. aureus* V8, a serine protease with a Z-score of 39.7 and 59% primary sequence identity (PDB code 1QY6) (33).

##### Structural Comparison of Esp and V8

The distances between Nϵ of the Esp active site His^117^ and Oγ of Ser^235^ and between Nδ of His^117^ and Oδ of Asp^159^ are 2.8 and 2.6 Å, respectively. Such short distances between the catalytic residues and their relative positions are conserved among canonical serine protease structures (56). A serine residue that forms a hydrogen bond with the catalytic Asp^159^ is sometimes referred to as the fourth member of the catalytic triad of serine proteases (57); however, this residue is absent in both Esp and V8; the corresponding space is occupied by Tyr^251^ and Trp^253^, respectively. Interestingly, the highly conserved hydrophobic residue (Trp or Phe) at position 215 of the serine protease family has been replaced by a Gly (Gly^252^ or Gly^254^) in both Esp and V8.

A key element of serine protease catalytic activity is the oxyanion hole, which is contributed by the right side wall of the S1 pocket (54). The oxyanion hole stabilizes the tetrahedral transition intermediate of the substrate scissile peptide bond by compensating the negative charge generated on its carbonyl oxygen during acylation (54). The oxyanion hole is formed by the backbone peptide nitrogen atoms of the catalytic Ser and two preceding residues in all serine proteases. The oxyanion hole is flexible and disordered in the zymogen (pro-forms) serine proteases. The conformation of Ser^235^-Asn^234^-Gly^233^-Gly^232^-Val^231^ peptide segment in Esp is rigid with conserved backbone angles, supporting the presence of a functional oxyanion hole. Further, the carbonyl oxygen of Gly^232^ in Esp is suitably pointing outwards and away from the hydroxyl group of the catalytic Ser^235^. With a distance of 4.2 Å between them, the catalytic residue Ser^235^ is positioned for a nucleophilic attack on the substrate scissile peptide bond's carbonyl carbon.

##### The Substrate-binding Pocket of Esp

Perona and Craik (54) defined seven conserved loops (loops A, B, C, D, 1, 2, and 3) in serine proteases that surround the S1 pocket (Fig. 8*b*), which exhibits high specificity toward the substrate P1 residue. Structural integrity of loop 1 has a direct impact on the catalytic efficiency of the enzyme, whereas loop 2 residue composition dictates the substrate P1 residue specificity and its binding efficiency. Variations in lengths and compositions of loop 3 as well as loops A, B, C, and D dictate the residue identity of the substrate at more distal positions on both sides of the P1 residue (54). Compared with all other eukaryotic serine proteases, loops 3 and D are absent in Esp and V8, and their loops 1 and 2 are also considerably shorter. A single polar residue on loop 1 (Asp in trypsin), at the bottom of the S1 pocket, dictates primary substrate specificity for serine proteases. We observe two polar residues, Ser^229^ and Thr^230^, at the loop 1 position of Esp (V8 Ser^231^ and Thr^232^). Nevertheless, these residues are not suitably positioned for interaction with the P1 residue bound in the S1 pocket. Using molecular modeling, Prasad *et al.* (33) suggested that Asn^258^, positioned in loop 2 of V8 (Esp Asn^256^) and pointing into the active site, may be the determinant of substrate specificity.

## DISCUSSION

When grown in liquid culture without rotation, many bacterial pathogens, including *S. aureus*, form biofilms on solid surfaces or at liquid-air interfaces (58). Research over the past two decades has identified bacterial genes and mechanisms supporting biofilm growth, which can be thought of as a developmental program with three or more discrete steps (59, 60). Biofilm formation initially requires bacterial adhesion to solid surfaces, and this includes bacterial adhesion to surfaces at liquid-air interfaces (61). Bacterial replication into a biofilm is dependent on cell-cell adhesions and on the establishment of an extracellular matrix, which is often comprised of DNA released from a subpopulation of biofilm bacteria but may also involve the synthesis of extracellular polysaccharides (61). Eventually, biofilms must release planktonic cells for dissemination in tissues of an infected host and/or development of new biofilm structures (60). These paradigms also appear to apply to the biofilms formed by *S. aureus* (62), a pathogen that colonizes human nares (1).

Iwase et al. (7) reported that *S. epidermidis* Esp, a secreted serine protease, can disperse *S. aureus* biofilms. Furthermore, colonization with Esp^+^ *S. epidermidis* strains was associated with protection from *S. aureus* colonization and administration of Esp^+^ *S. epidermidis* into the nares of human volunteers diminished *S. aureus* colonization (7). These findings provide strong support for the model of *S. aureus* biofilm formation in human nares; however, others have challenged this view and proposed that *S. aureus* may replicate as planktonic bacteria in the nasal cavity (63, 64).

Here we investigated the molecular basis of *S. epidermidis* Esp interference with *S. aureus* biofilm formation. Our data suggest that Atl is the premier target of Esp-mediated biofilm interference. Esp treatment diminished Atl-dependent release of extracellular DNA by cleaving the AM and GL murein hydrolase activities. Esp treatment did not affect biofilm formation for *atl* and *sspA* mutants of *S. aureus* Newman. X-ray crystallography revealed the three-dimensional structure of Esp, which is highly similar to that of *S. aureus* V8 (SspA) (33). V8 protease also cleaved Atl AM and GL and blocked biofilm formation. These data suggest that *S. aureus* biofilms are formed under conditions of controlled secretion and proteolysis of autolysin, a determinant for the release of DNA biofilm matrix. This developmental program can be perturbed by the Esp protease of *S. epidermidis* and by the V8 protease.

Earlier work reported that *sspA* expression in *S. aureus* SH1000, a variant of laboratory strain 8325-4 (RN6390B) in which the *rsbU* mutational lesion has been repaired (65), is required for biofilm formation when this strain is grown in 2% tryptic soy broth supplemented with 0.2% glucose but not when the strain is grown in TSB alone (52). In *S. aureus* SH1000, mutations in *sspA* and in other genes for extracellular serine proteases (*splABCDEF*) trigger a relative increase in extracellular protease activity (52, 66), which is associated with a reduction in biofilm formation. This phenotype is abolished in a genetic background where the structural gene for aureolysin (*aur*) has been deleted (52); aureolysin is a metalloproteinase that, following secretion into the extracellular medium, activates the serine proteases of *S. aureus* via removal of their pro-peptides (67, 68). Presumably, a cascade of secretion reactions and the sequential activation of extracellular proteases (aureolysin > cysteine proteases > serine proteases) control the activity of secreted Atl and the assembly or disassembly of staphylococcal biofilms (62).

We also solved the three-dimensional structure of Esp. A search for structural homologues of Esp using the DALI server (69) identified seven structures with less than 2.0 Å root mean square deviation value. *S. aureus* V8 protease was the best fit with a Z-score of 39.7 and 59% primary sequence identity (PDB code 1QY6) (33). Staphylococcal epidermolytic toxin A (ETA) (PDB code 1AGJ) was the second best with Z-score of 29.9 and 28% sequence identity (70). Staphylococcal secreted serine proteases SplB (PDB code 1VID) and SplA (PDB code 2W7U) displayed 32 and 28% identity, respectively (71). Glutamyl-endopeptidase (PDB code 1P3C, 26% identity) and exfoliative toxin B (PDB code 1QTF, 29% identity), with root mean square deviation less than 2 Å were also identified (72).

Nemoto and co-workers (73, 74) characterized *S. aureus* glutamyl endopeptidase V8 and identified, in addition to catalytic Ser^237^ (Esp Ser^235^), the N-terminal Val^69^ (Esp Val^67^) residue as essential for substrate cleavage. *S. aureus* V8 protease with an N-terminal truncation to Ile^70^ (Esp Ile^68^) was inactive, and mutants with an altered N-terminal residue Val^69^ (even with conserved substitutions) were also inactive, which is indicative of a strict requirement of the N-terminal Val residue for enzyme activity (75). Crystal structures of Esp and V8 display identical disposition for their N termini, which associate with respective S1 pockets more intimately than other active serine proteases (Fig. 8*c*). The N-terminal segment of Esp and V8 crosses over loop 1 into the bottom of the S1 pocket, and the N-terminal Val^67^ (V8 Val^69^) amino group is suitably positioned to act as an acceptor of the negative charge of P1 residue side chain (33). The N-terminal Val^67^ in Esp is positioned with its α-amino group located adjacent to the conserved Thr^230^ (V8 Thr^232^) and Asn^259^ (V8 Asn^261^), pointed into S1 pocket, within hydrogen bonding distance. Similarly, the His^250^ (V8 His^252^) residue on loop 2, conserved among glutamyl endopeptidases (73), having hydrogen bonds with side chains of conserved Tyr^226^ (V8 Tyr^228^) and Thr^230^ (V8 Thr^232^), is also suitably positioned to interact with the substrate acidic P1 residue.

**FIGURE 9.**
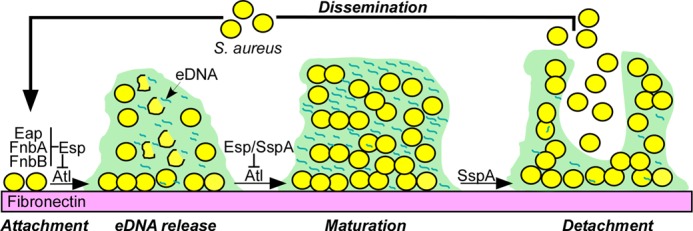
**Model illustrating *S. aureus atl*-dependent biofilm formation and the impact of serine proteases, *i.e.*, *S. epidermidis* Esp or *S. aureus* V8 (SspA), on controlling Atl activity and biofilm disassembly.** The model distinguishes five steps in the biofilm developmental process: attachment, eDNA release, maturation, detachment, and dissemination. Three surface proteins (Eap, FnbA, and FnbB) are thought to promote *S. aureus* attachment to fibronectin (attachment). The secretion of Atl promotes the release of eDNA as an extracellular matrix for biofilm formation (eDNA release). Activation of secreted SspA (V8 protease) inactivates Atl, thereby promoting staphylococcal replication in the newly formed matrix (biofilm maturation). The continued activation of SspA promotes the detachment of staphylococcal cells from the biofilm (detachment). Detached staphylococci disseminate and adhere elsewhere by binding to fibronectin and establishing another biofilm. *S. aureus* biofilm formation is perturbed by the *S. epidermidis* secreted protease Esp. We propose that exuberant expression of *S. epidermidis* Esp (unlike *S. aureus* SspA) perturbs biofilm formation of *S. aureus*.

Nevertheless, there are some notable differences between V8 and Esp in and around their S1 pockets (Fig. 8*d*) that can be associated with differences in substrate specificity. Extensive mutational analysis of V8 and Esp by Nemoto *et al.* (73) localized the difference in their specificities to Tyr^251^ (V8 Trp^253^) and Asp^255^ (V8 Pro^257^) residues on Esp loop 2. Substitutions at these positions affected mainly the *K_m_* with constant *k*_cat_ values, suggesting that these residues affect only substrate binding affinities (73). The *K_m_* value of native Esp harboring Tyr^251^-Val^254^-Asp^255^ on loop 2 was larger than that of V8 with Trp^253^-Val^256^-Pro^257^, but with almost similar *k*_cat_ values (76). Tyr^251^ of Esp is hydrogen-bonded with the side chain of Glu^223^, which is replaced by Ala^225^ in V8. In addition, the Lys^257^-Tyr^258^-Asn^259^-Ser^260^-Ser^261^ segment on loop 2 of Esp is replaced by Glu^259^-Tyr^260^-Asn^261^-Gly^262^-Ala^263^ in V8, all side chains pointing out of the S1 pocket, but into the known specificity determining secondary sites of serine proteases. Other notable residue differences between Esp and V8 in the vicinity of the S1 pocket include Tyr^92^ (V8 Gln^94^) and Tyr^99^ (V8 Thr^101^) on loop A. Thus, the S1 pockets of Esp and V8 preferentially bind negatively charged substrate side chains that are held in place by the amino group of the N-terminal Val^67^ (V8 Val^69^) residue and stabilized by conserved Thr^230^, His^250^, and Asn^259^ in Esp. However, the specificity differences between equally efficient Esp and V8 enzymes toward acidic P1 residue could be assigned to the differences observed in loop 2, specially to the Asp^255^ (V8 Pro^257^) present at the bottom of S1 pocket and pointing toward the catalytic site and other secondary residues on either side of the substrate P1 residue to the difference in loop 2 and loop D segments. These features of staphylococcal serine proteases may explain why Esp and V8 are able to cleave multiple domains of Atl and, when added exogenously during the early stages of biofilm formation, can interfere with the establishment of these structures. The V8 protease does contribute to biofilm formation of *S. aureus* Newman presumably by controlling the autolytic activity of Atl-derived AM and GL enzymes. Thus, secreted serine proteases can be viewed as biofilm regulatory factors that impact the production of biofilm matrix and the release of planktonic bacteria to initiate invasive disease (Fig. 9). If so, application of serine proteases (Esp or V8) as a treatment of nasal colonization with *S. aureus* may disperse planktonic staphylococci with invasive disease potential.

## Footnotes

2 The abbreviations used are:

Atl: *Staphylococcus aureus* autolysin
AM: Atl derived *N*-acetylmuramoyl-l-alanine amidase
Esp: *S. epidermidis* extracellular serine protease
GL: Atl derived glucosaminidase
PI: propidium iodide
R1, R2, and R3: repeat domains 1, 2, and 3, respectively, of *S. aureus* Atl
TSB: tryptic soy broth
PDB: Protein Data Bank.
